# Intestinal Parasitic Infections among Prison Inmates and Tobacco Farm Workers in Shewa Robit, North-Central Ethiopia

**DOI:** 10.1371/journal.pone.0099559

**Published:** 2014-06-13

**Authors:** Hassen Mamo

**Affiliations:** Department of Microbial, Cellular and Molecular Biology, College of Natural Sciences, Addis Ababa University, Addis Ababa, Ethiopia; UCSD, United States of America

## Abstract

**Background:**

Intestinal parasitic infections (IPIs) particularly soil-transmitted helminthiasis (STH) and schistosomiasis are among neglected tropical diseases (NTDs) globally. Apart from being associated with anemia, malabsorption and retarded cognitive development these diseases are complicating the clinical picture of more serious infections like HIV, TB and malaria. Renewed and up-to-date information on the epidemiology of IPIs in more vulnerable groups such as irrigated-farm workers and prisoners would significantly contribute towards improving the health condition of such at-risk groups.

**Methods:**

A cross-sectional survey was conducted to determine the prevalence of IPIs among prison inmates and tobacco farm workers in Shewa-Robit, north-central Ethiopia in November 2008. A total of 236 fecal samples were examined microscopically to detect helminths and/or protozoa using direct-smear and formol-ether concentration methods.

**Results:**

Overall, 8 intestinal parasite species have been recovered singly or in combinations from 146 (61.8 %) samples. The prevalence in prison population (88/121 = 72.7%) was significantly higher than that in tobacco farm (58/115 = 50.4%). There were no significant differences in the prevalence of IPI by most socio-demographics. Except for hookworm there was no significant difference in parasite prevalence between different age-groups though the frequency of individual parasites slightly varied between the age-groups. Multivariate logistic regression analysis showed that inmates were more likely to acquire IPIs than tobacco-farm workers (Odds Ratio (OR)  = 2.62, 95% confidence interval (CI)  = 1.52–4.5). In addition, participants who did not report past treatment for IPIs were more likely to acquire IPIs than participants who self-reported treatment for IPIs in the past twelve months (OR = 3.25, 95% CI = 1.75–6.06). All other socio-demographics were not significantly associated with IPIs in univariate analysis. *Entamoeba histolytica/dispar/moshkovskii* was the most frequently encountered species in both study sites accounting for 48.8 and 51.7 percent of the positives in prison and tobacco farm population respectively. Other intestinal parasites detected, with slight variation in prevalence in the two study areas, were hookworm, *Ascaris lumbricoides, Trichuris trichiura*, S*chistosoma mansoni*, *Strongyloides stercolaris*, *Hymenolepis nana* and *Taenia sp*. 35.5 and 33.0 percent of the total positive cases were mixed infections in Shewa Robit prison and tobacco farm, respectively.

**Conclusion:**

The results show that IPIs are common health problems in the studied populations.

## Introduction

A huge number of people suffer from intestinal parasitic infections (IPIs) especially soil-transmitted helminthiasis (STHs), the main species being *Ascaris lumbricoides*, *Trichuris trichiura* and the hookworms [1]. In 2010 alone; 819.0, 464.6 and 438.9 million people were infected with *A. lumbricoides*, *T. trichiura* and hookworm, respectively [2]. IPIs greatly affect the socio-economic development of communities in multiple ways [3], [4]. From health perspective, IPIs affect the physical and mental wellbeing of schoolchildren thereby leading to increased absenteeism, retarded cognitive development and thus learning disabilities [5], [6] although the literature is inconclusive [7]. Most importantly, IPIs complicate the clinical picture of more serious diseases like HIV/AIDS [8], [9] and malaria [10].

Eco-climatic conditions, geo-locality, socioeconomic, environmental, behavioral, cultural and demographic factors (particularly age) influence the prevalence of infection with different types of intestinal parasites [11]–[15]. An effective control of IPIs/Neglected tropical Diseases (NTDs) is believed to have a direct contribution to the Millennium Objectives for Development in least-developed nations. There is a need to continuously screen endemic communities to reduce the burden of IPIs as the WHO recommends periodic de-worming of IPIs, twice per annum if prevalence is over 50% and once if prevalence exceeds 20% [16]. Especially; more vulnerable groups, because of various additional socioeconomic, political or behavioral factors, require special attention. Occupational risk factors such as irrigated-farming make individuals living under such circumstances more vulnerable to IPIs compared to the general population. The risk of acquiring infectious or noninfectious diseases, including poor mental health, or activation and aggravation of already existing illness usually increases in prisons for various reasons.

Inmates with existing health problems do not received proper medical attention and may leave prisons in worse conditions than their previous life [17]. Although prisons in high-income nations have inmate healthcare systems the medical services in many prisons are commented as inadequate, underfunded, and understaffed, with common prisoners' abuse and mistreatment since prison populations are largely from poor minority communities that experience greater rates of chronic illness, substance abuse, and mental illness than the general population [18]. Infectious diseases in general and IPIs in particular are concerns of prison health because inmates are susceptible to disease through poor healthcare, overcrowding, demographics, high-risk behaviors, low-level immunity due to stress and inadequate or poor nutritional quality, and overall low-living standards compared to the general population [19].

As a result, prisoners carry a much greater burden of illness than other members of the society. Prison life is characterized by cascades of problems like severe drug abuse, alcoholism, trauma, homicide and suicide; and infectious diseases including intestinal parasites [20]. The occurrence of IPIs in institutions for mentally retarded and prisoners was higher (41.3–51.5%), compared to the general population, even in industrialized cities such as Helsinki [21]. 27.16% intestinal parasite prevalence was recorded among inmates in Canada [22]. A study conducted in the Madrid area has also revealed the relevance of IPIs within a prison population, presenting some differences to the situation in the general population [23]. Undoubtedly, in Africa the prevalence of IPIs in the prison population is high reaching over 70% [24]. The Ethiopian Shewa Robit prison where inmates from diverse ethnic backgrounds and locations living under similarly overcrowded and poor sanitary condition needed investigation.

Shewa Robit is a district town (Kewet District; North Shewa Zone) in the Amhara regional state some 225 km north of Addis Ababa where one of the four prisons under the Ethiopian Federal Prison Administration is found. The current sanitary status of the prison is poor as the other three federal prisons all built in the 1950s [25]. As it is the case with any other prison in Ethiopia Shewa Robit prison is a pool type, where one room housing several prisoners (approximately less than 2 m^2^ space per inmate). Also, Shewa Robit is the largest settlement in the district [26]. The town has a tobacco farmland where the indigenous and settled populations work. Farm workers are at a greater risk of exposure to intestinal parasites because of occupational risk. Irrigated-farm land is an ideal ground for the transmission of intestinal parasites [27].

Previous studies undertaken in Ethiopia revealed high prevalence of IPIs across the country [28]. But little information is available on the magnitude of intestinal parasites problem in vulnerable groups like prison inmates and irrigated farmland workers in and around Shewa Robit. Thus, the objective of the current study was to bridge this gap. The study is expected to help healthcare providers and concerned administrators make informed decision in resource mobilization and design of appropriate control strategies.

## Materials and Methods

### Ethics Statement

Institutional ethical clearance was obtained from the former Department of Biology Bioethics Review Committee, Addis Ababa University. Prior to sample collection participants were informed clearly about the objective and procedure of the study. Potential participants were told that participation was totally voluntarily, without the slightest negative consequence, and samples would be collected when they fully agreed by signing an informed consent. It was also explained to a potential participant that there was no foreseeable risk or undesirable side effect during fecal sample collection and any information obtained would remain confidential. Participants who were screened for intestinal parasites and were found infected by the same were treated free of charge as per standard treatment guideline. In general the author has followed best practices in publication and research ethics.

### Study area and population

Samples for the study were acquired from Shewa Robit prison and tobacco leaf development farm. Detailed description of Shewa Robit was reported elsewhere [29]. Before sampling concerned authorities were contacted and a request for cooperation was made after explaining the objective of the study. Prison inmates and farm workers in the respective areas were invited to participate in the study. Since there was no previous prevalence estimate in the area and prison population a sample size of 384 could have been attained using the formula 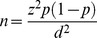, assuming 50% intestinal parasite prevalence; and 95% confidence interval with 5% marginal error [30]. At the time of sample collection the prison housed some 500 inmates. Given this small total source population it would have been possible to include the entire population. But 250 individuals were randomly chosen out of the total. However, among those asked to voluntarily submit stool specimen only 121 inmates complied limiting the sample size. That means a quarter of the prison population was sampled which was enough to represent the population. Tobacco farm workers including family members were about 203 and more than half (115) consenting individuals were sampled. The proximity of the prison to the tobacco farm makes the two populations comparable eco-epidemiologically. Similarly the two populations had their own risks. All age- and sex-groups in the area (settlers including inmates and indigenous) belonging to various ethnic groups volunteered to participate in the study. Socio-demographic data such as age, gender, duration of stay in the area and travel history were documented administering a semi-structured questionnaire. Information was also gathered on the history of IPIs and treatment for the same. An informational document about the study, including how to supply a stool specimen was given to each participant.

Stool specimens (about 0.5–1.5 g) were collected from 236 individuals in pre-labeled, leak-free, plastic specimen cups. The fecal specimens were physically examined and distinguished whether diarrheic (liquid/watery) or normal (well-formed samples). Diarrheic samples were examined immediately. Formed specimens were preserved in 15 ml of 10% formalin and transported to the biomedical laboratory, former Department of Biology, Addis Ababa University; Addis Ababa. The samples were examined using light microscopy for the presence of ova, larvae or cysts of intestinal parasites by direct smear and formalin-ethyl acetate sedimentation techniques [31].

### Data analysis

SPSS version 17.0 statistical package was used for data analysis. Differences in the proportions of IPIs between different groups were tested using the Chi-squared test. Further, univariate and multivariate logistic regression models were used to determine the predictive power of age, type of residence in the study area (settler/indigenous), ethnic group (Amhara/non-Amhara), duration of stay, travel history, past history of IPIs, past history of treatment for IPIs and study site in acquiring IPIs. The independent effect of the different variables in having IPIs was tested using odds ratios (OR) and 95% confidence intervals (CI) after adjustments. The differences were considered significant when the *p*-value was <0.05.

## Results

A total of 236 samples were collected from inmates (n = 121) and tobacco farm workers (n = 115). Table 1 shows age and sex frequencies of the study population. The study population was mainly composed of the Amhara (68.6%). Other smaller groups were Oromo 25 (10.6%), Tigre 12 (5.1), Wolaita 11 (4.7%), Gamo 9 (3.8%), Gurage 4 (1.7%), Silte 4 (1.7%), Keficho 4 (1.7%), Berta 1 (0.4%), Dawuro 1 (0.4%), Hadya 1 (0.4%), Kambata 1 (0.4%) and Schekicho 1 (0.4%). Of these, 91 (38.6%) individuals were indigenous Amharas and 145 (61.4%) were non-indigenous, most non-Amhara and some Amharas. During sample collection, 14 inmates had resided in the prison for less than a year. Overall, there were 145 individuals who had resided in the study locality for more than three years (61.4% of the total group interviewed). The mean length of duration in the study locality for inmates was 2.5 years and for non-indigenous/settler workers was 13 years. Of the participants interviewed, 65% gave a verbal history of taking repeated therapy for IPIs at different times in the past. But it was not possible to determine for which parasites they were treated or which drugs were used in their therapy.

**Table 1:** Demographic characteristic of study participants from Shewa Robit prison and tobacco farm.

Study site	Age groups	Male (n, %)	Female (n, %)	Total (n, %)
Prison (n = 121)	15–24	64(52.8)	3 (2.4)	67 (55.2)
	25–34	34 (28.1)	0 (0.0)	34 (28.1)
	≥35	20 (16.5)	0 (0.0)	20 (16.5)
Tobacco farm (n = 115)	15–24	30 (26.1)	6 (5.2)	36 (31.3)
	25–34	28 (24.3)	10 (8.7)	38 (33.0)
	≥35	33 (28.6)	8 (7.0)	41 (35.6)

n =  number of volunteers, % =  percentage.

Overall, 146 individuals (61.8%) were infected with one or more intestinal parasites belonging to eight different species or types: *A. lumbricoides*, *E. histolytica/dispar/moshkovskii*, hookworm, *Hymenolepis nana, Schistosoma mansoni, Strongyloides stercoralis, Taenia sp, T. trichiura*. While 55.4% of the cases were mixed infections (48.8% among prison population and 65.5% in tobacco farm) the rest were single (table 2). The most commonly encountered parasite in both study populations was *E. histolytica/dispar/moshkovskii*, 48.8% of all infections in Shewa Robit prison and 51.1% in tobacco farm. The second most common infection in the prison population was hookworm and in tobacco farm it was *A. lumbricoides*. The other frequently encountered parasite was *T. trichiura*. In general the prevalence of intestinal parasites in the prison population (88/121 = 72.7%) was significantly higher than that in the tobacco farm (58/115 = 50.4%) (*p*  = 0.03).

**Table 2:** Rate of intestinal parasite single and mixed infections out of total positive cases in Shewa Robit prison (88) and tobacco farm (58).

Parasite species	Study site	
	
**Single infections**	**Prison, n (%)**	**Tobacco farm, n (%)**
*E. histolytica/dispar/moshkovskii*	17 (19.3)	7 (12.0)
*A. lumbricoides*	8 (9.0)	4 (6.8)
Hookworm	6 (6.8)	2 (2.2)
*S. mansoni*	0 (0.0)	3 (5.1)
*T. trichiura*	7 (7.9)	2 (3.4)
*S. stercoralis*	5 (5.6)	1 (1.7)
*H. nana*	2 (2.7)	1 (1.7)
Total	45 (51.1)*	20 (34.4)*
**Mixed infections**		
*E. histolytica/dispar/moshkovskii/A. lumbricoides*	7 (7.9)	7 (12.0)
*E. histolytica/dispar/moshkovskii/hookworm*	11 (12.5)	4 (6.8)
*E. histolytica/dispar/moshkovskii/S. mansoni*	0 (0.0)	1 (1.7)
*E. histolytica/dipar/moshkovskii/T. trichiura*	8 (9.0)	11 (18.9)
Hookworm/*A. lumbricoides*	9 (10.2)	9 (15.5)
Hookworm/*Taenia species*	5 (5.6)	1 (1.7)
Hookworm/*S. mansoni/T. trichiura*	0 (0.0)	1 (1.7)
*S. stercoralis/T. trichiura*	3 (3.4)	4 (6.8)
Total	43 (48.8)**	38 (65.5)**

*While 34.4% of total positive cases were single infections in Shewa Robit tobacco, 51.1% were single infections among inmates.

**48.8% and 65.5% of total positive cases were mixed infections in prison and tobacco farm, respectively.

The distribution of individual parasites with age-group for Shewa Robit prison and tobacco farm was indicated in tables 3 and 4. Except for hookworm there was no significant difference in parasite prevalence between the different age-groups though the frequency of individual parasites slightly varied between the age-groups. In both prison and tobacco farm the prevalence of hookworm was significantly lower in the age-group 15-24 years old compared with those above 24 (*p*<0.001). *A. lumbricoides* and *E. histolytica/dispar/moshkovskii* were found relatively more frequently in the 15- to 24-years age-group in both study populations.

**Table 3:** Distribution of intestinal parasites among different age groups of Shewa Robit prison inmates.

Parasite species	Age groups (years)			
	15–24 (n = 67)	24–34 (n = 34)	≥35 (n = 20)	Total
*A. lumbricoides*	13 (19.4)	6 (17.6)	5 (25.0)	24 (19.8)
Hookworm	4 (5.9)	9 (26.4)	14 (70.0)	27 (22.3)
*E. hist/dispar/moshkovskii*	23 (34.3)	12 (35.2)	8 (40.0)	43 (29.7)
*H. nana*	1 (1.4)	0 (0.0)	1 (5.0)	2 (1.6)
*T. trichiura*	7 (10.4)	6 (17.6)	5 (25.0)	18 (14.8)
*S. stercoralis*	1 (1.4)	1 (2.9)	3 (15.0)	5 (4.1)
*Taenia species*	2 (2.9)	2 (5.8)	1 (5.0)	5 (4.1)
*S. mansoni*	0 (0.0)	0 (0.0)	0 (0.0)	0 (0.0)

**Table 4:** Distribution of intestinal parasites among different age groups of Shewa Robit tobacco farm workers.

Parasite species	Age groups (years)			
	15–24 (n = 36)	24–34 (n = 38)	≥35 (n = 41)	Total
*A. lumbricoides*	9 (25.0)	8 (21.0)	3 (7.3)	20 (17.3)
Hookworm	2 (8.3)	3 (10.5)	12 (29.3)	17 (14.7)
*E. hist/dispar/moshkovskii*	10 (27.7)	11(28.9)	9 (21.9)	30 (26.8)
*H. nana*	0 (0.0)	0 (0.0)	1 (2.4)	1 (0.86)
*T. trichiura*	8 (22.2)	4 (10.5)	6 (14.6)	18 (15.6)
*S. stercoralis*	0 (0.0)	3 (7.8)	2 (4.8)	5 (4.3)
*Taenia species*	0 (0.0)	0 (0.0)	1 (2.4)	1 (0.86)
*S. mansoni*	1 (2.7)	2 (5.2)	2 (4.8)	5 (4.3)

There were no significant differences in the prevalence of IPIs by most socio-demographics. Multivariate logistic regression analysis showed that geo-locality and self-reported past history of treatment for IPIs are independently associated with acquiring IPIs after adjustment. Inmates were more likely to acquire IPIs than tobacco farm workers (OR = 2.62, 95% CI = 1.52–4.5). In addition, participants who did not report past treatment for IPIs were more likely to have IPIs than participants who self-reported treatment for IPIs in the past twelve months (OR = 3.25, 95% CI = 1.75–6.06) (table 5). All other socio-demographics were not significantly associated with IPIs in univariate analysis. The number of infected females and diarrheic individuals was too low to perform statistical test and were omitted from analysis.

**Table 5:** Univariate and multivariate analysis of socio-demographics and associations with IPIs in Shewa Robit prison and tobacco farm.

Socio-demographics	IPI^1^ Prevalence no (%)	total examined (n = 236)	Odds Ratio (95% CI^2^)	*p*-value
**Age (years)**				
14–24	67(65.0)	103	0.78(0.5–1.3)	0.41
≥25	79(59.3)	133		
**Gender** †				
*Male*	131(62.6)	209		
*Female*	15(55.6)	27		
**Residence status**				
*Indigenous*	55(56.0)	91	1.10(0.64–1.9)	0.78
*Settler*	91(62.7)	145		
**Ethnic group**				
*Amhara*	103(63.5)	162	0.79(0.45–1.39)	0.47
*Non-Amhara*	43(58.1)	74		
**Site**				
*SRT* 3	58(50.4)	115	2.62(1.52–4.5)	<0.001
*SRP* 4	88(72.7)	121		
**Duration of stay**				
*≤3 yrs*	51(56.0)	91	1.44(0.84–2.47)	0.21
*>3 yrs*	94(64.8)	145		
**Travelled out of SR** 5				
*Yes*	103(60.5)	170	1.21(0.67–2.19)	0.55
*No*	43(65.1)	66		
**Self-reported IPI (past 12 months)**				
*Yes*	92(58.9)	156	1.44(0.82–2.54)	0.21
*No*	54(67.5)	80		
**Self-reported treatment for IPI (past 12 months)**				
*Yes*	83(53.2)	156	3.25(1.75–6.06)	<0.001
*No*	63(78.7)	80		
**Diarrhea during sample collection** †				
*Yes*	2(28.5)	7		
*No*	141(61.5)	229		

1 *IPI*: intestinal parasite infection;

2 *CI*: confidence interval;

3 *SRT*: Shewa Robit tobacco farm;

4 *SRP*: Shewa Robit prison;

5 *SR*: Shewa Robit;

† Sample size for female and diarrheic participants was too small to perform any statistical test.

## Discussion

The increased rate of IPIs among the inmates is similar to reports from elsewhere in Africa [32]. The higher rate of feco-orally transmitted infections like *A. lumbricoides* and *E. histolytica/dispar/*moshkovskii indicates dissemination of these infections under institutional conditions. However, A. lumbricoides and Entamoeba differ a bit in their transmission. Although both orally, protozoa are immediately infectious, where eggs of Ascaris may need a while to become infective. Because of this reason autoinfection may be common for Entamoeba. Once infected, individuals may indefinitely propagate the protozoa unless treated. Also, even if hygienic facilities improve in the two institutions it might be impossible to clean the participants from preexisting infection at home, especially Entamoeba. This may explain the most common occurrence of entamoeba among the study populations in the present study. Contamination of drinking water with Entamoeba sp has been increasingly recognized as a cause of water-borne human disease worldwide [33].

61.4% of the total interviewed reported that they had resided in the study locality for more than three years. In sub-Saharan African settings most prisons lack basic healthcare facilities including proper record systems. So it was difficult to cross-check the self-reported data. During sample collection almost all participants were asymptomatic and had no diarrhea. When intestinal discomforts are felt it is not uncommon to take metronidazole or mebendazole/albendazole, at least some aware and financially able people, from private drug vendors. Therefore, the significantly higher IPIs in individuals who reported no treatment history in the past twelve months, prior to sampling, compared to those who reported an intervention may suggest that in some individuals the treatment was effective and no immediate re-infections followed. It also may indicate the level of reliability of the self-reported data. Nonetheless, self-reported data should be treated with caution. It is possible that self-reports of infections and treatment may be subject to social desirability biases. Moreover, the possibility of recall-bias is there. The likelihood of losing the detail of an infection and its treatment due to memory-decay even in literate people is high [34]. This problem may result in the inclusion of infection/its symptom that might have occurred outside the period of interest.

Although confounding factors may not always be corrected for, IPIs showed significant variation between different ethnic or tribal groups in certain settings [35]. But the current study could not reveal association between presence of IPIs and ethnicity suggesting lack of genetic predisposition for the examined parasites. Similarly, unlike the report from a Nigerian prison where the distribution of parasites based on the duration of imprisonment [36] the present study has shown no variation in IPIs with duration of stay suggesting that inmates get IPIs irrespective of their arrival time in the prison. But the possibility of acquiring these infections by the inmates before coming to the prison cannot be ruled out. Initiating de-worming schemes for newly arriving inmates and sustaining the chemotherapy in the prison may reduce parasite propagation inside the prison.

Varying prevalence rates, the highest ever being 93%, have been reported in Ethiopia with variations for individual parasites [37], [38]. It is true that there is heterogeneity in the reports on intestinal parasite prevalence in Ethiopia. The possible explanations for the observed discrepancies might be attributed to variations in study design, possible differences in the quality of water source and other sanitary facilities, and local customs or behavior. Differences in the method of stool detection, climate of the study area, study season, experience of screening technicians and overall quality control procedures may account for the apparent differences in the prevalence of intestinal parasites across the country.

The time of the study also matters. Some of extreme prevalence values [38] were older reports which may not be comparable to the situation now in the country. Nowadays there are significant improvements in the level of community awareness about personal and environmental hygiene and parasite transmission. Enhanced outreach strategy and extensive health extension services including periodic deworming schemes have contributed towards decline of IPIs in the general population. Changing patterns are being noticed in the prevalence of intestinal parasites as it is the case elsewhere in Africa [39]. For instance, King et al. 2013 [40] reported 24.2% prevalence which is considerably lower compared to a previous study in 1995 [41] which recorded 48.5% prevalence of STH from the same setting in Ethiopia. Similarly from the southwestern part of the country a lower prevalence (39%) was recorded [42]. The present overall prevalence rate in the prison population is above the findings of most recent community-based studies in the country suggesting the more vulnerable situation of the prison population.

*S. mansoni* has a wide range of distribution occurring in several parts of Ethiopia [43]. In this study five cases of *S. mansoni* infections were identified, all from tobacco farm. The workers were engaged in cultivating tobacco leaf using irrigation water. Activities and situations like irrigation, swimming and fishing are critical in schistosomiasis epidemiology. One of schistosome-positive individuals was not born in the study area but had grown up and had been living there for a long period of time. However, the individual had travelled to Kemissie (to the north of the study area) which is a well-known schistosomiasis endemic focus in Ethiopia [43]. This might suggest that the individual had acquired the infection during her recent travel to the endemic area since the presence of local transmission remained uninvestigated. Another schistosme-infected individual was indigenous and travelled only to Goba where from no cases of schistosome infections have so far been reported. The three remaining infected individuals had no travel history outside Shewa Robit confirming the presence of local transmission of *S. mansoni*.

There is no report on IPIs in general and intestinal schistosomiasis in particular from Shewa Robit and its surroundings apart from one report more than 30 years back [44] which documented some cases of *S. mansoni*. However, the study did not reveal whether those cases were because of indigenous transmission or imported ones. Thus, the present study is the first of its kind to report endemic *S. mansoni* from Shewa Robit. But the epidemiology of intestinal schistosomiasis in Shewa Robit especially among tobacco farm workers requires further investigation. Wider and more comprehensive future studies including malacological surveys are required to establish the endemicity of intestinal schistosomiasis in the area.

*Ascaris* and *Trichuris* are the most common coexisting geo-helminths widely distributed in Ethiopia with increased prevalence rate at higher altitudes [37]. The high rate of hookworm cases is indicative of lack of protective clothing tradition including shoe-wearing and thus easily skin exposure among community members. The significantly higher hookworm infection rate among the older groups may be explained by certain occupational and behavioral risk factors. Most adult community members are engaged in cultivation and other related activities which expose them to parasites like schistosomes, hookworm and *S. stercolaris*. *S. stercolaris* coexists with hookworm in the same altitudinal range. It was reported from many parts of Ethiopia with the exception of very arid and semi-arid areas [44]. Further, adults freely move from garden to garden which increases the risk of infection by various parasite species. Although no evidence is available in the present study, the practice of using ‘night soil’ (human feces) for soil fertility is among factors that expose adult farmers to IPIs elsewhere [45].

In general the detection of a wide range of intestinal parasites in the study population is a reflection of the poor environmental sanitation and personal hygienic practices. Sanitary facilities in the homes/workplaces may be inadequate to the majority of the people. The relatively crowded living condition and composition of inmates who were almost from all regions of Ethiopia could create a fertile ground for intestinal parasite transmission. Equally, the migration of people from central and northern parts of Ethiopia to the area seeking job may be identified as an important epidemiological factor for the introduction of a wide range of intestinal parasites.

## Conclusions

Intestinal parasitism in general and STH in particular is an important public health problem in Shewa Robit prison and tobacco farm. The most commonly encountered parasites in the study have the potential to cause anemia and malabsorption and other complications. Therefore, routine examination of stool samples and treatment of infected individuals on regular basis would significantly contribute towards improving the health condition of irrigated-farm workers and inmates thereby reducing the burden of IPIs in the wider population. Intervention strategies including health education on personal hygiene are required. This is especially emphasized in relation to the public health importance of NTDs in individuals with HIV/AIDS. A comprehensive investigation of intestinal parasites in the region, with emphasis on the epidemiology of schistosomiasis is warranted.
